# The Internalization of Social Stigma Among Minor-Attracted Persons: Implications for Treatment

**DOI:** 10.1007/s10508-019-01569-x

**Published:** 2020-01-10

**Authors:** Rebecca Lievesley, Craig A. Harper, Helen Elliott

**Affiliations:** 1 Department of Psychology, Nottingham Trent University, 50 Shakespeare Street, Nottingham, NG1 4FQ UK; 2 School of Social Sciences, Bishop Grosseteste University, Lincoln, UK

**Keywords:** Minor-attracted persons, Stigma, Sexual abuse prevention, Well-being, Pedophilia

## Abstract

In this article, we sought to build on existing stigmatization research by examining the extent to which internalized stigmatization (i.e., the personal adoption and incorporation of social views, operationalized as thought suppression—an avoidant coping strategy—and low psychological well-being) among minor-attracted persons (MAPs) may impact upon help-seeking behaviors and their avoidance of children. We adopted a cross-sectional anonymous survey design to recruit a sample of self-identified MAPs (*N* = 183) from prominent online support fora. We found that increased levels of suppression and lower levels of psychological well-being were associated with lower levels of hope about the future, but higher levels of both shame and guilt about having a sexual interest in minors. Thought suppression was not significantly associated with outcomes related to help-seeking behaviors, but did significantly predict higher rates of actively avoiding children, even after controlling for psychological well-being and other emotional variables. Independently, lower levels of self-reported psychological well-being were associated with a desire for more support and higher rates of actively avoiding children. We explore the potential implications of our data in relation to treating and supporting MAPs within the community, increasing their well-being, and encouraging help-seeking behavior.

## Introduction

Minor attraction is a topic associated with widespread social debate and controversy (Seto, 2008). While pedophilia (sexual attraction to prepubescent children) is the most well-known and widely studied chronophilic category related to minor attraction, other categories involving a sexual attraction to children also exist (Blanchard et al., 2009; Seto, 2017), including hebephilia (sexual attraction to pubescent children) and ephebophilia (sexual attraction to postpubescent adolescent minors). In this article, we are interested in exploring the experiences of those individuals whose sexual orientation (Seto, 2012) is dominated by their sexual attraction toward children, regardless of their preferred or predominant age of attraction, and thus, the broader term “minor-attracted persons” (MAPs) is adopted to account for this. We may refer to “pedophilia” in places, however, as this has been used as a catch-all term for minor attraction in both popular and academic discourses (Harper & Hogue, 2015; King & Roberts, 2017).

While sexual interests toward minors and other paraphilias have been identified as key risk factors for sexual (re-)offending (Hanson & Morton-Bourgon, 2005; Helmus, Ó Ciardha, & Seto, 2015), contemporary research in Germany has reported that fewer than half of all individuals convicted of child abuse offenses actually have a pedophilic sexual preference (Schmidt, Mokros, & Banse, 2013). Further, there is an unknown number of individuals with pedophilia as their predominant sexual arousal pattern (as well as MAPs with other ages of attraction) in the community, who never commit any offenses at all (Cantor & McPhail, 2016). Some estimates place the specific prevalence of pedophilia within the general (male) population at between < .1–5% (Dombert et al., 2016; Seto, 2008). However, other researchers have found much higher rates of minor attraction when looking at sexual attraction to “children” in a more general sense. For example, around 10% of German men engage with sexual fantasies involving children (Ahlers et al., 2011), while one-in-four men would seemingly continue to sexualize an online conversation with somebody who reveals themselves as a child (Bergen, Antfolk, Jern, Alanko, & Santtila, 2013). These statistics indicate that low-level sexual interests in children may be widespread within the general population.

Against the popular conflation of “pedophile” (and “MAP”) with “child molester” (Feelgood & Hoyer, 2008; Harrison, Manning, & McCartan, 2010), there are several online communities of self-identified MAPs who engage with and support one another to prevent child sexual abuse and who engage with the general public in an effort to reduce this common misunderstanding (Holt, Blevins, & Burkert, 2010). In spite of this, those who are labeled as pedophiles (either accurately as a result of either their assessed or self-reported sexual orientation, or incorrectly as a result of some form of sexual offense against a child) face large amounts of stigmatization (Harper, Bartels, & Hogue, 2018; Imhoff, 2015; Jahnke, 2018a, 2018b; Jahnke & Hoyer, 2013; Jahnke, Imhoff, & Hoyer, 2015a). In this article, we build on this stigmatization research, by examining the extent to which thought suppression (a proxy measure of the internalization of social stigma) among MAPs may impact upon help-seeking behaviors and active attempts to avoid coming into contact with children.

### The Stigmatization of Minor-Attracted Persons

Stigmatization is the process of forming negative evaluations of an individual or groups of people based on limited characteristics (Corrigan, Morris, Michaels, Rafacz, & Rusch, 2012). It is typically discussed in relation to three distinct but interrelated domains. First, stigmatization possesses an emotional component. This may be linked to negative automatic feelings about in individual or group based on their membership of that population (Harper et al., 2018; Vess, 2009). Second, stigmatization involves a cognitive process, whereby those who are responsible for doing the stigmatizing make specific (negative) attributions about the stigmatized group. In relation to people with sexual interests in minors, these attributions may involve ascribing choice to the sexual orientation, or making a presumption of offending behavior (Imhoff, 2015; for a comprehensive review, see Jahnke, 2018b). Third, there is a behavioral component to stigmatization, which may include the support for punitive policy positions in relation to a stigmatized group, or a desire to obtain social distance from them (Jahnke et al., 2015a).

While a growing amount of research is emerging in relation to the social stigmatization of MAPs (e.g. Harper et al., 2018; Imhoff, 2015; Imhoff & Jahnke, 2018; Jahnke, 2018a; Jahnke et al., 2015a), our focus is on the transference of this into self-stigmatization, MAP well-being, and help-seeking behavior. The self-stigmatization that takes place within the MAP community has been reported as being associated with an exaggerated fear of discovery, as well as reductions in cognitive and emotional functioning (Jahnke, Schmidt, Geradt, & Hoyer, 2015b). These deficits include significant associations between having a MAP identity and having a heightened fear of discovery, perceived social distance from the rest of the population, lower levels of self-esteem, and higher levels of subjective social isolation (Jahnke et al., 2015b). Not only do these issues link to well-being, but they are also implicated as potential risk factors for the commission of sexual offenses (see e.g., Gillespie, Mitchell, Fisher, & Beech, 2012). Having more psychosocial issues, a history of mental health problems, and difficulties in controlling one’s sexual attractions were all recently reported as being associated with sexual offending in a community sample of MAPs (Cohen, Ndukwe, Yaseen, & Galynker, 2018; though see Neutze, Seto, Schaefer, Mundt, & Beier, 2011), who found relatively few differences between those who have and have not acted on their attractions to minors. However, the social isolation of MAPs has also been linked to a lack of willingness to actively seek therapeutic support for these sexual interests (Jahnke & Hoyer, 2013), potentially compounding both low levels of well-being and further distancing this group from services aimed at both improving MAP well-being and preventing child sexual abuse. In their open-ended questionnaire study, Grady, Levenson, Mesias, Kavanagh, and Charles (2019) reported social stigma and a fear of discovery as the most important barriers to help-seeking behavior among MAPs, with internalized stigma (i.e., seeing themselves as a “bad person” because of their unchosen sexual attractions Grady et al., 2019) and a fear of being reported to legal authorities also being prominent themes in participant narratives. Jahnke (2018b) also sets out a series of recommendations for professionals working with MAPs. While Jahnke reported that a large proportion of people who are predominantly attracted to minors (i.e., their minor attraction is more than their attraction to adults) had thought about seeking professional help, there is commonly either a lack of willingness to treat this group (Koops, Turner, Jahnke, Märker, & Briken, 2016; Steils-Glenn, 2010) or a mismatch between therapist and MAPs treatment goals (for results from a non-peer-reviewed survey of MAPs on this topic, see B4U-ACT, 2011). These data suggest that perceptions of stigma (and, in some cases, internalized stigma) can act as a barrier to help-seeking and access to professional support services.

#### Internalized Stigma in Minor-Attracted Persons

According to the literature within social psychology, members of stigmatized and marginalized social groups experience high levels of stress. The minority stress model (Meyer, 2003) offers a theoretical framework for understanding this and builds on seminal theorizing on the intra- and interpersonal effects of prejudice and experienced stigma (Allport, 1954; Goffman, 1963; Link & Phelan, 2001). This model was developed after an examination of the experiences and effects of such stigmatization and purports minority stress to be unique (i.e., it is additive to normal everyday stresses that are experienced by everybody), chronic (i.e., it is stable over time), and rooted in established social structures, norms, and hierarchies. Meyer (2003) proposed that three processes are at play when considering minorities’ experiences of trauma, stress, or prejudice within society. First, there is either a chronic or acute experience. In the case of MAPs, this could be a physical attack (an acute event), or the continued experience of hate within society via the media (a chronic event; see Jahnke et al., 2015a). Second, there is a process of expectation, whereby minorities begin to anticipate such negative experiences and become hyper-vigilant and sensitive to potential stressors. Third, there is some degree of internalization of social stigmatization, which is commonly accompanied by concealment activities. These strategies have been observed in homosexual (Mohr & Daly, 2008) and transgender populations (Beemyn & Rankin, 2011). The internalization of negative social attitudes, stereotypes, and attributions is what we refer to as the experience of internalized stigma and has been observed among these groups (e.g., Hendricks & Testa, 2012), with this being posited as a motivation to engage in stress-ameliorating strategies in the form of distancing oneself from the minority identity via concealment (hiding one’s identity from those external to oneself), and suppression (refusing to accept one’s own minority identity).

An interaction between concealment and internalization could lead some minority groups to actively attempt to suppress their identities. That is, suppression may act as a form of self-concealment, such as to ameliorate minority stress and distance the minority individual from the negative feelings that they may have begun to internalize about themselves. Examples of groups in which thought suppression has been viewed as a method of identity (self-)concealment includes women who have had abortions (Major & Gramzow, 1999) and members of the LGBT community (Crocker & Major, 1989; Hendricks & Testa, 2012). Although Jahnke (2018b) described how this process could work in relation to MAPs (i.e., social stigma leading to attempts to conceal one’s MAP identity), no study has previously examined suppression efforts within the MAP community in a direct or empirical manner.

While this process of thought suppression can be a positive strategy for alleviating acute experiences of stigma in the short term, there is the potential that this strategy could be counterproductive in the longer term. For example, Smart and Wegner (2000) describe many cognitive burdens that are associated with the constant suppression of one’s identity. Having to maintain an outward appearance that is different to internal processes can lead to a preoccupation with suppressing (Meyer, 2003), which can lead the individual living an internal “private hell” (Smart & Wegner, 2000, p. 229). This may be especially the case among MAPs who, even when in private, have no legal way of acting on their sexual attractions, meaning that suppression could become a totalizing experience in all areas of life with profoundly negative effects on psychological well-being. This is particularly troubling in light of evidence of a potential rebound effect that is associated with suppressing unwanted thoughts. Erskine and Georgiou (2011) described a range of evidence, suggesting that trying not to think about particular things (e.g., memories or actions) may actually increase rumination on those topics and reduce self-regulation processes (see also Abramowitz, Tolin, & Street, 2001). For example, dieters who suppress thoughts of hunger or thirst have been found to eat less in the short term, but binge at a later time (Denzler, Förster, Liberman, & Rozenman, 2010; Erskine, 2008), while the same outcomes are observed among smokers trying to reduce their cigarette consumption (Erskine, Georgiou, & Kvavilashvili, 2010). In the sexual domain, suppressing sexual thoughts has been associated with higher levels of compulsive sexual behaviors in religious groups (Efrati, 2019). The implications in relation to MAPs are stark in this regard. That is, if increased levels of suppression are associated with a rebound effect in relation to increased engagement in the thoughts and behaviors being suppressed, then thought suppression related to minor attraction could paradoxically increase a propensity to engage with sexual thoughts involving children.

While the above commentary suggests that thought suppression (along with an associated index of psychological well-being) acts as a direct proxy measure of internalized stigma, there may be other explanations as to why MAPs would suppress their sexual attractions. For example, it could be that experiences of societal stigma (and the reduced levels of well-being that are associated with this; Jahnke et al., 2015b) leads some MAPs suppress their sexual thoughts. While suppressing sexual thoughts about children helps some MAPs to function better in their everyday lives and avoid ruminating on their sexual interests and does not, in itself, reflect internalized stigma, the conscious effort that this suppression takes does reflect some degree of not wishing to experience those sexual thoughts. In doing so, MAPs who suppress for this reason are internally “admitting” that there is something wrong with their sexual thoughts—even in the absence of offending behavior. This indirectly does indicate a form of internalized stigma, as experiencing a sexual thought is subjectively different to causing harm by acting upon it. The former can be experienced in a healthy way and alleviated without causing harm (e.g., via fantasy engagement and masturbation), while the latter causes harm at both the individual and societal levels (for a meta-analysis of the effects of child sexual abuse, see Paolucci, Genius, & Violato, 2001). In suppressing sexual attractions that can be expressed in a healthy way, MAPs may themselves conflate their attractions with something more sinister, thus internalizing the stigma that they face within society in a manner consistent with Meyer’s (2003) minority stress model.

Taking this further, it may be that this internalization of social stigma has subsequent effects on help-seeking behaviors. As reported previously, Grady et al. (2019) found that MAPs who viewed themselves as inherently bad people (they referred to this as shame in relation to sexual attractions) were less likely to seek help from professionals for issues arising from their attractions to minors. If we adopt the view that suppression represents the internalization of social stigma, and this stigma leads to shame and social withdrawal (Jahnke et al., 2015b), we might expect higher levels of thought suppression to be associated with a lesser willingness to seek professional support, even if this suppression is associated with increased shame and worse emotional well-being.

### The Present Study

In this study, we sought to examine the role of internalized social stigma in relation to help-seeking behavior among an online community of self-identifying MAPs. We used suppression of unwanted thoughts as a proxy measure of internalized social stigma about a sexual interest in minors for a number of reasons. First, suppression is associated with the active avoidance of unwanted thoughts (Rassin, 2003)—a process commonly observed among stigmatized groups demonstrating higher levels of minority stress (Meyer, 2003). Given that sexual cognitions and fleeting sexual themes are common in intrusive thoughts (Cathey & Wetterneck, 2013), and that having sexual interests in minors is a highly socially stigmatized issue (Jahnke & Hoyer, 2013), we start with the assumption that sexual thoughts (and associated reminders of the social stigma related to their sexual orientation) will be prevalent among samples of MAPs. However, by asking about the suppression of unwanted thoughts more generally (as measured using the White Bear Suppression Inventory [WBSI; Wegner & Zanakos, 1994]), we can examine this idea in a more subtle manner without potential demand characteristics associated with explicitly priming thoughts of a minor-attracted identity. This is also consistent with associated research in other areas of clinical psychology, which have also used the WBSI to examine levels of thought suppression in patients with mental health issues such as depression (Thimm, Wang, Waterloo, Eisemann, & Halvorsen, 2018), obsessive–compulsive disorder (Ching & Williams, 2018), and schizophrenia-related symptoms (Jones & Fernyhough, 2009) At the same time, the advertising of our survey (i.e., a research project hosted on a website for self-identified MAPs) may subjectively or implicitly prime participants to consider their responses within the broader context of their status as MAPs.

We predicted that high levels of suppression would be positively associated with feelings of shame and guilt about their sexual interests in minors, but negatively associated with hope for the future. We also predicted that suppression would be negatively associated with a general measure of psychological well-being (Hypothesis 1). Further, we predicted that those who had sought help for their sexual interests would be less likely to suppress unwanted thoughts than those who had not previously sought support (Hypothesis 2). We also expected suppression to be associated with both a desire for more support with their sexual interests (Hypothesis 3) and a propensity to engage in the active avoidance of children (Hypothesis 4).

## Method

### Participants

A total of 183 participants (*M*_age_ = 33.17 years, *SD* = 12.09) who self-identified as being attracted to minors were recruited through social media and online forums/support organizations. Specific demographic information is presented in Table 1, though the sample was predominantly male (90%), white (95%), and single (68%). In terms of age of attraction, we used Blanchard et al.’s (2009) criteria to categorize participants as having a pedophilic attraction pattern if they were attracted to minors aged 10 years of age or below (*n* = 23; 12.5%), as having an (ep)hebephilic^1^ attraction pattern if they were attracted to minors aged 11–16 years (*n* = 23; 12.5%), and as having a paedo(ep)hebephilic attraction pattern if their age of attraction included both of these age ranges (*n* = 137; 75%).The majority of participants were exclusively attracted to minors (73%). Table 1 also presents suppression scores for each demographic group, though no formal analyses are conducted on these data due to vastly unequal sample sizes.

**Table 1:** Sample demographic information and WBSI score

Demographic characteristic	*n* (%)	WBSI suppression score
*Sex*		
Male	165 (90%)	47.12 ± 13.45
Female	10 (6%)	63.20 ± 7.27
Age	33.17 ± 12.09	*r *= − .20, *p* = .008
*Ethnicity*		
White	174 (95%)	47.68 ± 13.64
Black	1 (< 1%)	69.00 ± 00.00
Hispanic/Latino/a	6 (3%)	45.83 ± 16.61
Native	1 (< 1%)	71.00 ± 00.00
Other	1 (< 1%)	51.00 ± 00.00
*Relationship status*		
Single	124 (68%)	47.73 ± 12.97
In a relationship	58 (32%)	48.40 ± 15.54
*Parent*		
Yes	26 (14%)	44.31 ± 13.25
No	157 (86%)	48.48 ± 13.83
*Employment status*		
Full-time employed	84 (46%)	43.81 ± 14.41
Part-time employed	21 (12%)	55.10 ± 11.63
Student	39 (21%)	52.79 ± 11.28
Out of work (looking)	18 (10%)	46.83 ± 12.91
Out of work (not looking)	5 (3%)	55.80 ± 13.14
Volunteer	2 (1%)	57.00 ± 4.24
Retired	4 (2%)	48.00 ± 12.78
Unable to work	9 (5%)	54.56 ± 11.26
Highest qualification	5.60 ± 1.19	*r *= − .20, *p* = .006
*Sexual orientation*		
Heterosexual	63 (34%)	48.11 ± 12.21
Homosexual	37 (20%)	44.57 ± 11.56
Bisexual	44 (24%)	52.11 ± 16.60
Other	39 (21%)	45.90 ± 13.90
*Chronophilic grouping*		
Paedophilic	23 (13%)	47.57 ± 14.74
(Ep)hebephilic	23 (13%)	45.04 ± 15.79
Paedo(ep)hebephilic	137 (75%)	48.42 ± 13.31
*Exclusive MAP*		
Yes	133 (73%)	47.28 ± 13.45
No	50 (27%)	49.50 ± 14.65

Percentages may not always equal 100% due to missing data (when lower than 100%) and rounding (when above 100%). WBSI = White Bear Suppression Inventory (scoring range 15–75; higher scores are equal to more suppression; data represent means ± one SD). Data for age (years) and education (1–7 ordinal scale; higher scores are equal to more education) are means ± one SD (column one), and zero-order correlations with WBSI scores (column two)

### Procedure

The research was advertised on a number of English-speaking online forums/support organizations for individuals with a sexual interest in minors and was available in English only. Information regarding exactly where the survey was advertised is not reported here, as forum anonymity was specified within the consent procedures to offer an additional layer of identity protection for participants and organizations. The administrators of the organizations and forums were approached by the research team and agreed to advertise the research on our behalf. Additionally, the research was advertised on social media platforms which allowed for the sharing of the research across a wider online community of MAPs.

The advert provided a brief overview of the study and a link to access the survey. The online survey was developed using Qualtrics and did not record traceable information (e.g., IP addresses) in order to provide total anonymity for potential participants. Those interested in taking part followed the link and were taken to the first page of the survey which provided detailed information about the study and their participation. Those who wished to proceed were required to provide consent after reading the provided information by clicking a button that then directed them to the full survey. From there, participants completed the survey which took approximately 20–30 min and were then provided with debrief information which reiterated the purpose of the study, their right to withdraw, a number of support options, and the researchers contact details and thanked them for their participation. The withdrawal process was via email, where participants had to state their unique, anonymous identifier (created at commencement of the survey). No participants withdrew from this research.

### Measures

#### Demographics

Participants were asked to respond to a range of demographic questions at the beginning of the study. These included questions about their sex, age, occupation, education, sexual interests, and current relationship status. This information was only collected for descriptive purposes.

#### White Bear Suppression Inventory (WBSI; Wegner & Zanakos, 1994)

The WBSI is a 15-item self-report measure that examines respondents’ suppression of unwanted thoughts. Each item (e.g., “I often do things to distract myself from my thoughts”) is rated using a 5-point scale (1 = strongly disagree, 5 = strongly agree), with responses summed into a composite suppression score. Thus, the potential scoring range spans 15–75, with high scores indicating high levels of suppression. The WBSI demonstrated excellent internal consistency in the present sample (α = .92; *n* = 183).

#### Warwick-Edinburgh Mental Well-being Scale (WEMWBS; Tennant et al., 2007)

The 14-item WEMWBS was used to measure participants’ subjective levels of psychological well-being. This scale asks respondents to consider how they have been feeling in the previous 2 weeks and identify the extent to which they have experienced particular states. Each state (e.g., “I have been dealing with problems well”) is then responded to using a 5-point scale (1 = none of the time, 5 = all of the time). Scores for each response are then summed to leave a potential range of 14–70, with higher scores indicating greater subjective levels of well-being. The WEMWBS demonstrated excellent levels of internal consistency in the current sample (α = .93; *n* = 179).

#### Personal Feelings Questionnaire Version 2 (PFQ-2; Harder & Zalma, 1990)

The PFQ-2 measures shame (e.g., embarrassment) and guilt (e.g., remorse) by examining how often a responder experiences a range of emotions. In the present study, participants were asked to rate these emotions specifically in relation to their sexual interests in minors. A total of 22 emotions were listed. Of these, ten measured shame, six measured guilt and a further six were “filler” emotions (e.g., enjoyment). Each emotion listed on the PFQ-2 is rated using a 5-point scale (0 = never experience the feeling, 4 = experience the feeling continuously, or almost continuously). This means that the total scoring ranges are 0–40 for shame and 0–24 for guilt. The PFQ-2 demonstrated very good levels of internal consistency in the present sample (shame α = .88; guilt α = .82; both *n* = 82).

#### The Herth Hope Index (HHI; Herth, 1992)

The HHI is an adapted version of the Herth Hope Scale (HHS). The measure consists of 12 items (e.g., “I believe that each day has potential”), each of which is rated using a 4-point scale (1 = strongly disagree, 4 = strongly agree). Thus, the scoring range spans from 12 to 48, with high scores indicating increased levels of hope about the future. The HHI demonstrated excellent internal consistency in the present sample (α = .90; *n* = 178).

#### Brief Self-Control Scale (Tangney, Baumeister, & Boone, 2004)

This 13-tem measure of state self-control asked participants to rate themselves against a series of statements (e.g. “I am good at resisting temptation”) using a 5-point scale (1 = not at all, 5 = very much). Scoring thus ranges from 13 to 45, with high scores indicating higher levels of self-perceived self-control. This scale demonstrated very good levels of internal consistency in the present sample (α = .84; *n* = 178).

#### Brief COPE Scale (Carver, 1997)

The 28-item COPE scale was used to measure participants’ use of a range of different coping strategies. While the original structure of the COPE purports to measure 14 different strategies, we used Scnider, Elhai, and Gray’s (2007) three-factor revised structure, identifying “problem-focused” (8 items, e.g., “I’ve been taking action to try to make the situation better”; α = .78), “active emotional” (10 items, e.g., “I’ve been getting emotional support from others”; α = .76), and “avoidant emotional” strategy clusters (10 items, e.g., “I’ve been giving up trying to deal with it”; α = .61). Each potential coping strategy was rated on a 4-point scale (1 = I haven’t been doing this at all, 4 = I’ve been doing this a lot), and scores were summed for each subscale. A total of 179 participants completed this measure.

#### Active Avoidance of Children

Participants were asked to select one response from a five-point ordinal measure of active child avoidance. The response options for this question were as follows: (1) “I never feel the need to avoid children,” (2) “Not often,” (3) “Occasionally,” (4) “Some of the time,” and (5) “All of the time.” All 183 participants answered this question.

#### Help-seeking Behavior

Participants were asked to state whether they had previously sought help for their sexual interests (Yes/No). Additionally, participants were asked to rate their current level of perceived support in relation to their sexual interests. Three response options were available: (1) “I feel I can manage by myself,” (2) “I feel I have enough support,” and (3) “I feel I need further support.” A total of 181 participants answered both questions.

## Results

We analyzed our data in four ways. We first investigated the relationships between our measured variables using correlational analyses. We then examined whether we could discriminate between whether an individual had previously sought help for their sexual interests using the range of variables measured, before examining whether these factors were associated with (1) whether more support for their sexual interests was desired and (2) whether our participants actively avoided children.

### Correlational Analyses

We ran correlations on each of our measured variables in order to examine their relationships. Pearson correlation coefficients and descriptive statistics are shown in Table 2.

**Table 2:** Zero-order correlations and descriptive statistics on measured variables

	1	2	3	4	5	6	7	8	9	10	11	12
1. WBSI	–	− .39***	.43***	.50***	− .38***	− .04	.05	.58***	− .46***	− .05	.36***	.36***
2. Hope		–	− .28*	− .21	.82**	.55***	.43***	− .51***	.51***	.06	− .18*	− .19*
3. Guilt			–	.78	− .36**	− .21	− .19	.31*	− .24	.06	.27*	.55***
4. Shame				–	− .34*	− .02	.15	.47***	− .10	− .04	.30*	.41**
5. WEMWBS					–	.43***	.43***	− .44***	.40***	− .01	− .26***	− .27***
6. COPE—PFC						–	.45***	− .20**	− .24**	.08	.29***	.07
7. COPE—AcEC							–	.01	− .13	.01	.23**	.10
8. COPE—AvEC								–	.22**	.12	.13	− .02
9. BSCS									–	.00	− .25**	− .20**
10. Past help-seeking										–	.21**	.04
11. Need more support											–	.23**
12. Active child avoidance												–
*M*	47.89	32.30	10.70	15.15	43.05	20.39	25.96	19.80	40.42	.46	2.17	2.00
SD	13.79	7.57	7.16	8.37	11.42	4.81	5.34	5.28	9.22	.50	.82	1.28
Scoring range	15–75	12–48	0–24	0–40	14–70	8–32	10–40	10–40	13–45	–	1–3	1–5

WBSI = White Bear Suppression Inventory; WEMWBS = Warwick-Edinburgh Mental Well-being Scale; PFC = problem-focused coping; AcEC = active emotional coping; AvEC = avoidant emotional coping; BSCS = Brief Self-Control Scale. Past Help-Seeking = dummy-coded (0 = no, 1 = yes)

*p < .05; **p < .01; ***p < .001

As expected, higher scores on the WBSI (indicating increased levels of thought suppression) were associated with higher levels of guilt and shame about participants sexual interests, lower levels of hope about the future, and lower levels of subjective psychological well-being. However, it was also associated with higher levels of avoidant emotional coping. These correlations indicate that thought suppression is not only linked to a range of deleterious effects in relation to indices of emotion and mental health, but also this conscious process may be associated with more avoidant approaches to problem solving, which is important among MAPs within the context of professionals attempted to encourage help-seeking. In relation to the key outcome variables, suppression was unrelated to past help-seeking behavior but was positively correlated with a desire for more support than was currently being received. Further, suppression was positively correlated with a higher incidence of actively avoiding children. These results are consistent with Hypothesis 1.

Further, we explored the level of thought suppression within the current sample in order to compare these levels to those observed in other clinical populations.^2^ In the present sample the average WBSI score was 47.89 ± 13.79. This is comparable to or higher than WBSI scores reported among patients with depression (*M* = 39.10 ± 9.94, *t*[211] = 3.35, *p* = .001, *d* = 0.73; Thimm et al., 2018), disordered eating habits (*M* = 45.21 ± 12.84, *t*[392] = 2.00, *p* = .047, *d* = 0.20; Ferreira, Palmeira, Trindade, & Catarino, 2015), and obsessive–compulsive disorder (*M* = 47.65 ± 12.72, *t*[301] = 0.15, *p* = .878, *d* = 0.02; Ching & Williams, 2018). Interestingly, the current sample’s WBSI scores were also significantly higher than those reported for undergraduate males in non-clinical settings (*M *= 44.30 ± 10.80, *t*[336] = 2.63, *p* = .009, *d* = 0.29; Rassin, 2003). These comparisons suggest that thought suppression may be at least as significant a feature of being minor-attracted as it is in other clinical groups, and more prominent in this population than it is within the general community.

### Past Help-Seeking Behavior

We ran a binary logistic regression in order to establish whether our collection of measured variables could distinguish between membership of the “help-seeking” group (i.e., those participants who had previously sought professional support for their sexual interests, vs. those who had not). We measured the following variables: WBSI, shame- and guilt-proneness, hope, WEMWBS, and problem-focused, active emotional, and avoidant emotional coping strategies. We also controlled for age, relationship status (single vs. in a relationship), parental status (has children vs. does not), and education level. However, shame- and guilt-proneness and hope were collinear with scores on the WEMWBS (operationalized as VIF values > 3, violating suggested thresholds; Hair, Risher, Sarsedt, & Ringle, 2019). As such, we removed these three variables from the model.

Contrary to Hypothesis 2, the overall model was not statistically significant, $R^{2}_{N} = .125$, *χ*^2^(10) = 16.26, *p* = .092. This indicates that the model overall could not adequately distinguish between help-seekers and non-help-seekers (total correct classification = 67.5%). Only two demographic variables were uniquely associated with the “help-seeking” group membership. These were age (with older participants being more likely to have sought support; *Z* = 2.67, *p* = .008) and relationship status (with people in a relationship being more likely to have sought support; *Z* = 2.44; *p* = .015). All model coefficients are shown in Table 3.

**Table 3:** Binary logistic regression predicting “help-seeking” group membership

	*B*	SE	*Z*	*p*	OR	95% CI (OR)	
						Lower	Upper
Intercept	− .25	1.76	− .14	.888	.78	.03	24.61
WBSI	− .01	.02	− .89	.375	.99	.96	1.02
WEMWBS	− .01	.02	− .35	.728	.99	.96	1.03
COPE—PFC	.08	.05	1.68	.092	1.08	.99	1.18
COPE—AcEC	.00	.04	.08	.936	1.00	.93	1.08
COPE—AvEC	.00	.04	.07	.943	1.00	.92	1.09
BSCS	− .03	.02	− 1.33	.184	.97	.93	1.01
**Age**	**.05**	**.02**	**2.67**	**.008**	**1.05**	**1.01**	**1.08**
**Relationship status**	**.92**	**.04**	**2.44**	**.015**	**2.51**	**1.20**	**5.25**
Parental status	− .75	.52	− 1.45	.148	.47	.17	1.31
Education	.18	.16	− 1.16	.245	.84	.62	1.13

WBSI = White Bear Suppression Inventory; WEMWBS = Warwick-Edinburgh Mental Well-being Scale; PFC = problem-focused coping; AcEC = active emotional coping; AvEC = avoidant emotional coping; BSCS = Brief Self-Control Scale; Relationship status = dummy-coded (0 = single, 1 = in a relationship); Parental status = dummy-coded (0 = no children, 1 = children). Significant independent variables are presented in bold

### Desire for More Support

We next conducted a linear multiple regression to see whether our measured variables were associated with a desire for *more* support within our sample. As previously, shame- and guilt-proneness and hope were all collinear with the WEMWBS and were removed as variables within the model. This model was explained a significant proportion of the variance in desire for more support, $R^{2}_{\text{adj}} = .226$, *F*(10, 155) = 5.81, *p* < .001.

Within the model, lower psychological well-being (*β* = − .20, *p* = .037) was associated with a greater desire for more support in relation to managing sexual interests in children. In addition, those participants who were in a relationship (*β* = .25, *p* < .001) were more likely to state that they desired more support than they were currently receiving. While not directly supporting Hypothesis 3, these data do suggest that lower levels of well-being (which is associated with internalized stigma in the form of thought suppression) are associated with a desire for more support. Having a problem-focused approach to coping was also associated with an increased propensity to desire further support, though this fell just short of statistical significance (*β* = .17, *p* = .053). Full model coefficients are shown in Table 4.

**Table 4:** Multiple linear regression examining desire for further support

	*B*	SE	*t*	*p*	*β*	95% CI (*β*)	
						Lower	Upper
Intercept	1.76	.61	2.91	.004			
WBSI	.01	.01	1.56	.120	.15	− .04	.33
**WEMWBS**	− .**01**	**.01**	− **2.10**	**.037**	− .**20**	− .**39**	− .**01**
COPE—PFC	.03	.02	1.95	.053	.17	− .00	.34
COPE—AcEC	.00	.01	.29	.771	.03	− .14	.19
COPE—AvEC	.02	.01	1.59	.113	.15	− .04	.33
BSCS	− .01	.01	− 1.21	.227	− .11	− .28	.07
Age	.00	.01	.23	.82	.02	− .14	.18
**Relationship status**	**.43**	**.13**	**3.42**	**< .001**	**.25**	**.11**	**.40**
Parental status	.08	.17	.46	.65	.04	− .12	.19
Education	− .06	.05	− 1.23	.22	− .09	− .24	.06

WBSI = White Bear Suppression Inventory; WEMWBS = Warwick-Edinburgh Mental Well-being Scale; PFC = problem-focused coping; AcEC = active emotional coping; AvEC = avoidant emotional coping; BSCS = Brief Self-Control Scale; Relationship status = dummy-coded (0 = single, 1 = in a relationship); Parental status = dummy-coded (0 = no children, 1 = children). Significant independent variables are presented in bold

### Active Avoidance of Children

We repeated the above linear multiple regression with active avoidance of children as the criterion. Again the model explained a significant proportion of the variance in relation to this outcome, $R^{2}_{\text{adj}} = .149$, *F*(10, 157) = 3.93, *p* < .001.

Within this model, higher levels of thought suppression (*β* = .28, *p* = .004) and lower psychological well-being scores (*β* = − .21, *p* = .037) were significantly associated with a greater propensity for actively avoiding children. This is consistent with Hypothesis 4. Full model coefficients are shown in Table 5.

**Table 5:** Multiple linear regression examining levels of active avoidance of children

	*B*	SE	*t*	*p*	*β*	95% CI (*β*)	
						Lower	Upper
Intercept	1.76	.98	1.80	.075			
**WBSI**	**.03**	**.01**	**2.91**	**.004**	**.28**	**.09**	**.47**
**WEMWBS**	− .**02**	**.01**	− **2.10**	**.037**	− .**21**	− .**41**	− .**01**
COPE—PFC	.02	.02	.88	.381	.08	− .10	.26
COPE—AcEC	− .02	.02	− 1.11	.269	− .10	− .27	.08
COPE—AvEC	− .00	.02	− .05	.957	− .01	− .20	.19
BSCS	− .00	.01	− .17	.863	− .02	− .19	.16
Age	.00	.01	.14	.890	.01	− .15	.18
Relationship status	.37	.20	1.81	.072	.14	− .01	.29
Parental status	− .31	.28	− 1.10	.275	− .09	− .25	.07
Education	.03	.08	.33	.745	.03	− .13	.12

WBSI = White Bear Suppression Inventory; WEMWBS = Warwick-Edinburgh Mental Well-being Scale; PFC = problem-focused coping; AcEC = active emotional coping; AvEC = avoidant emotional coping; BSCS = Brief Self-Control Scale; Relationship status = dummy-coded (0 = single, 1 = in a relationship); Parental status = dummy-coded (0 = no children, 1 = children). Significant independent variables are presented in bold

## Discussion

### Summary of Key Findings

The aim of this study was to establish whether thought suppression and psychological well-being (proxies for the internalization of social stigma) among MAPs played a role in views about help-seeking and the active avoidance of children by members of this community.

Consistent with Hypothesis 1, we found that suppression was associated with higher levels of shame and guilt about being attracted to minors and lower levels of hope about the future. The opposite trends were true for psychological well-being. We can interpret these relationships as indicating some degree of internalization of social stigma, which aligns with prior work on the experience of stigma-related stress in this population (Jahnke et al., 2015b). That is, higher guilt and shame about the sexual orientation toward minors are consistent with the internal adoption of societal attitudes toward people with sexual interests in minors. Further, the links between these negative emotions and suppression suggest that this identity is often unwanted and is associated with lower levels of psychological well-being (as demonstrated by the negative correlation between thought suppression and the WEMWBS). The associations between suppression and well-being with hope, in particular, may indicate that those who have internalized social stigma to a greater extent also see themselves as being “trapped” by their sexual identity, with little chance of happiness as they move forward in life.

Contrary to Hypotheses 2 and 3, we did not find any differences between those who had or had not sought help for their sexual interests in minors in relation to their tendency to suppress unwanted feelings. This suggests that any potential internalization of social stigma, or any worries that an individual may have associated with this, is not necessarily associated with actual rates of help-seeking behavior. This is inconsistent with other research in relation to thought suppression and identity concealment in other sexual minority groups (Chaudoir & Fisher, 2010) and may suggest that levels of acceptance of the MAP identity (both by MAPs themselves and those from whom they seek help) are low even among those who disclose this information to others. However, we did find that those who reported feeling that they were in need of *more* support than they were currently receiving scored lower on the index of psychological well-being—a construct that is significantly associated with the suppression of unwanted thoughts in this population. This suggests that the need for therapeutic input and support may be greater among those who have begun to internalize the social stigma around having a sexual interest in minors.

Finally, consistent with Hypothesis 4, we found actively avoiding coming into contact with children was linked to both a tendency to suppress unwanted thoughts and feelings and lower levels of psychological well-being. This is arguably the most interesting (and yet the starkest) of our findings, as it may be suggestive of several things. Of most importance, the active avoidance of children among MAPs may be indicative of an increased self-perceived risk of offending behavior. That is, if somebody perceives that they may pose a risk to children (as is the prevailing social message portrayed about MAPs; Feelgood & Hoyer, 2008; Jahnke, 2018b), they may take steps to actively avoid coming into contact with them as an active method to reduce this. This is consistent with Maruna, LeBel, Naples, and Mitchell’s (2009) criminological theorizing of so-called “Golem effects,” whereby the low expectations of society turn into self-fulfilling prophecies among the targets of those expectations. An alternative interpretation of this result, and one with more empirical support from the literature as it currently stands (see Jahnke et al., 2015b), is that the active avoidance of children represents a defensive action designed to either (1) protect the individual from having their sexual attractions discovered and/or (2) prevent them from forming a close friendship with a child that could ultimately be lost upon the potential discovery of their MAP status. In both of these cases, the active avoidance of children appears to be reflective of some form of internalized stigmatization of participants’ MAP identities. That is, if avoidance reflects a self-perceived risk, then the social discourse that conflates minor attraction with child sexual abuse has become internalized, while if avoidance reflects a fear of discovery, perceptions of social difference (i.e., that MAPs are in some way different to non-MAPs) have become internalized.

### Implications for the Treatment of MAPs: Adopting a Health-Based Approach

Schemes that are designed to prevent or reduce the incidence of child sexual abuse (e.g., Germany’s Dunkelfeld Project) have been evaluated in relation to risk-relevant outcomes and dynamic sexual offense risk factors (e.g., pro-criminal attitudes, impression management, and delinquent behavior; Beier et al., 2009, 2015), with these not showing particularly the positive results (see Mokros & Banse, 2019). While this lack of “treatment success” may call into question the validity of such schemes, it may be that these schemes emerge as being much more successful if broader psychosocial constructs related to well-being were also assessed. Further, the risk-based framing of such schemes (i.e., “prevention projects”) implicitly assumes that MAPs need to be “prevented” from offending (Lievesley & Harper, 2019), rather than “supported” though their psychosocial experiences of being attracted to minors (Grady et al., 2019; Jahnke, 2018b; Jahnke et al., 2015b). This is a claim supported by self-reported experiences and expectations of MAPs when surveyed about what they believe therapist aims are (B4U-ACT, 2011) and may mean that those wishing to distance themselves from such a label do not reach out to access services that could lead to significant well-being improvements (Lievesley & Harper, 2019).

Not only does the framing of MAP support services have implications for help-seeking behavior, but there may also be subsequent effects on the internalization of social (and perceived professional) stigma. That is, when presented with hostility from the general community, and perceiving risk reduction and abuse prevention and the core aims of professional and clinical support services, MAPs may begin to take on this stigmatization and incorporate it into their own identities (Grady et al., 2019). This may go some way to explain the high levels of thought suppression within our sample, with WBSI scores among MAPs being higher than or equivalent to those observed in patients with depression, eating disorders, and obsessive–compulsive disorder (Ching & Williams, 2018; Ferreira, Palmeira, Trindale, & Catarino, 2015; Thimm et al., 2018). Further theoretical support for this conclusion comes from the minority stress model (Meyer, 2003) which cites identity concealment and internalized stigmatization as key facets of experiencing trauma as a direct consequence of having a minority identity. In having these effects, the very framing of prevention services thus may play a pivotal role, not in making society safer, but rather in serving to potentially increase the risk of offending among some MAPs by contributing to worse mental health (e.g., higher levels of guilt and shame and lower levels of hope about the future; see Cohen et al., 2018). This is particularly problematic in light of the evidence of thought suppression having a behavioral rebound effect in associated research (e.g., in relation to disordered eating, smoking, and compulsive sexual behavior; Denzler et al., 2010; Efrati, 2019; Erskine et al., 2010).

One potential therapeutic route to address these issues may be to encourage the acceptance of the minor attraction among the MAP community (Lievesley, Elliott, & Hocken, 2018), which is consistent with Cantor’s (2014) conceptualization of a more identity-positive approach to sexual abuse prevention. That is, the emphasis of treatment should be less related to “risk reduction” and more related to promoting MAPs’ social and psychological well-being, with forensic risk reduction becoming a by-product of this broader aim. Acceptance-based approaches to treatment have recently been recognized as an alternative to Cognitive Behavioral Therapy (CBT). Instead of taking a deficits-focused view of the problem at hand (i.e., adopting a risk-focused lens), acceptance-based approaches such as Acceptance and Commitment Therapy (ACT; Hayes, Strosahl, & Wilson, 1999; Hayes & Wilson, 1994) work with the idea that peoples’ difficulties occur when they attempt to avoid and suppress painful feelings (Hayes, 1994), and represent a more positive psychological approach to dealing with distress. As such, we recommend that treatment approaches tackle the negative self-image experienced by many MAPs by encouraging them to view their sexual interests as a core part of their identities (acceptance), but also support them to live crime-free lives (commitment). Treatment may thus be aligned with established principles within the area of sexual crime desistance, such as those embedded within the Good Lives Model (GLM; Ward & Brown, 2004) by encouraging therapists to assist service users in achieving primary human goods related to inner peace, rather than fixating on managing or changing their sexual attractions. While these approaches lack a comprehensive empirical evidence base at the time of writing, the emergence of new tools to measure key facets of the ACT (Bond et al., 2011) and GLM (Harper et al., 2019) frameworks offers researchers the opportunity to systematically explore these concepts and how they may be associated with MAP experiences.

### Limitations and Future Directions

As noted previously, we used proxy measures of stigma internalization and risk in the present study. That is, we used the tendency to suppress unwanted thoughts and an index of psychological well-being as indirect proxies of internalized stigma among MAPs, while self-reported frequency of actively avoiding children was used at the outset of the project as an indication of self-perceived risk of committing acts of sexual abuse (though see above for an alternative interpretation of this construct that still reflects the internalization of stigma). This strategy was used such as to not prime themes related to stigmatization and to not implicitly assume this population were at an enhanced risk of sexually offending against children (Cantor & McPhail, 2016). In addition, our question about help-seeking behaviors was non-specific, and so responses could include a combination of participants accessing professionally delivered schemes and more informal social supports. Examining any differences in “help-seeking” by this professional/personal division would be an interesting avenue for further research. Further, there were limitations placed upon us by the moderators of the forums that we worked with in order to gather the data (e.g., in asking about offending behaviors that participants may have engaged in). Nonetheless, these are limitations that future research should seek to address.

More explicit measures of the internalization of stigma, such as a modified version (such as to directly relate to the topic at hand) of the Internalized Stigma of Mental Illness scale (Ritsher, Otilingham, & Grajeles, 2003), could be incorporated. Self-perceived risk is a more difficult topic to assess indirectly. With the launch of new technologies allowing the embedding of indirect measures to online surveys (e.g., the iatgen applet extension, which facilitates the use of the implicit association test in surveys created using Qualtrics; Carpenter et al., 2019), it might be possible to examine implicit levels of acceptance of child–adult sexual relationships in a manner similar to how implicit judgements of rape cases have been examined (e.g., Harper, Franco, & Wills, 2019). Another more straightforward alternative would be to use Gannon and O’Connor’s (2011) Interest in Child Molestation scale, which explicitly asks respondents to rate (1) how aroused they might be if they were to put themselves in the position of a fictitious child sex offender, (2) whether they would do the same as this fictitious offender, and (3) the extent to which they would enjoy behaving in that manner. However, this method is fraught with potential limitations, not least the potential for socially desirable responding, and the enhanced risk of participant attrition.

Away from these methodological limitations, it would be useful to examine some of our recommendations about the potential utility of ACT-based therapeutic principles as a route to reducing self-stigmatization and perceived risk of committing acts of child sexual abuse. Existing support services, such as the Dunkelfeld Project, should shift their therapeutic focus to incorporate more acceptance-based strategies into their ongoing work at the local level in a small-scale pilot study. A thorough evaluation of any such pilot—both in terms of its ability to reduce offense-related constructs *and* improve MAP well-being—should be undertaken prior to more widespread adoption of these principles, but some form of small-scale test of this approach appears to be deserving of investigation.

### Conclusions

Here, we reported a cross-sectional study of self-identifying MAPs rates of suppression of unwanted thoughts and psychological well-being. Associated with these suppression strategies were high levels of shame and guilt in relation to their sexual interests in minors, low levels of hope about the future, and a propensity to actively avoid children. We believe that these all have implications for the treatment of those individuals with sexual interests in minors who come forward to receive support. It may be constructive for professionals working with this population to encourage the “ownership” of the minor-attracted sexual identity, such as to reduce levels of self-stigmatization and increase self-acceptance. In doing so, we argue that we (as professionals, and as a society) can empower MAPs to take control of their own destinies, work constructively toward living a “good life” (Ward & Brown, 2004), improve MAP well-being, and, ultimately, protect children from sexual harm by improving MAP well-being and agency.

## Footnotes

1 Here we use (ep)hebephilia as a catch-all term for those MAPs who did not report any paedophilic sexual attractions. While we acknowledge that Seto (2017) defines hebephilia and ephebephilia as specific chronophilic categories, subgroup sizes in our sample mean that combining these groups makes sense, particularly in light of a lack of specific planned analyses in relation to chronophilic grouping.

2 We thank an anonymous reviewer for this helpful suggestion, which allowed us to contextualise the level of suppression in the current sample in relation to those observed in other areas of clinical research.
